# Internet-Specific Epistemic Beliefs in Medicine and Intention to Use Evidence-Based Online Medical Databases Among Health Care Professionals: Cross-sectional Survey

**DOI:** 10.2196/20030

**Published:** 2021-03-18

**Authors:** Yen-Lin Chiu, Yu-Chen Lee, Chin-Chung Tsai

**Affiliations:** 1 Department and Graduate Institute of Medical Education and Bioethics National Taiwan University College of Medicine Taipei Taiwan; 2 Department of Chinese Medicine China Medical University Hospital Taichung Taiwan; 3 Graduate Institute of Acupuncture Science China Medical University Taichung Taiwan; 4 Program of Learning Sciences National Taiwan Normal University Taipei Taiwan; 5 Institute for Research Excellence in Learning Sciences National Taiwan Normal University Taipei Taiwan

**Keywords:** evidence-based medicine (EBM), health care professionals, internet-specific epistemic beliefs, medical informatics

## Abstract

**Background:**

Evidence-based medicine has been regarded as a prerequisite for ensuring health care quality. The increase in health care professionals’ adoption of web-based medical information and the lack of awareness of alternative access to evidence-based online resources suggest the need for an investigation of their information-searching behaviors of using evidence-based online medical databases.

**Objective:**

The main purposes of this study were to (1) modify and validate the internet-specific epistemic beliefs in medicine (ISEBM) questionnaire and (2) explore the associations between health care professionals’ demographics, ISEBM, and intention to use evidence-based online medical databases for clinical practice.

**Methods:**

Health care professionals in a university-affiliated teaching hospital were surveyed using the ISEBM questionnaire. The partial least squares-structural equation modeling was conducted to analyze the reliability and validity of ISEBM. Furthermore, the structural model was analyzed to examine the possible linkages between health professionals’ demographics, ISEBM, and intention to utilize the evidence-based online medical databases for clinical practice.

**Results:**

A total of 273 health care professionals with clinical working experience were surveyed. The results of the measurement model analysis indicated that all items had significant loadings ranging from 0.71 to 0.92 with satisfactory composite reliability values ranging from 0.87 to 0.94 and average variance explained values ranging from 0.70 to 0.84. The results of the structural relationship analysis revealed that the source of internet-based medical knowledge (path coefficient –0.26, *P*=.01) and justification of internet-based knowing in medicine (path coefficient 0.21, *P*=.001) were correlated with the intention to use evidence-based online medical databases. However, certainty and simplicity of internet-based medical knowledge were not. In addition, gender (path coefficient 0.12, *P*=.04) and academic degree (path coefficient 0.15, *P*=.004) were associated with intention to use evidence-based online medical databases for clinical practice.

**Conclusions:**

Advancing health care professionals’ ISEBM regarding source and justification may encourage them to retrieve valid medical information through evidence-based medical databases. Moreover, providing support for specific health care professionals (ie, females, without a master’s degree) may promote their intention to use certain databases for clinical practice.

## Introduction

### Evidence-Based Medicine

Evidence-based medicine, defined as an integration of the best available external evidence, individual clinical expertise, and patient preferences in making optimal decisions for patient care, remains a major concern for health care professionals, public health practitioners, and medical educators [1-3]. Applying the most efficacious treatment integrated with evidence-based medicine will maximize the quality and quantity of life of individual patients [1]. Practicing evidence-based medicine may promote rapid updating of knowledge, improve expected patient care, and have positive effects on doctor-patient relationships [4-6].

As the internet provides a broad range of web-based medical information for health care professionals in their daily practice, their web-based medical information–searching behaviors have become an issue of great interest [7,8]. The internet has become the most frequently used resource for obtaining medical information in clinical practice [9], while also being considered as an evidence-based medical tool [10]. Evidence-based online medical databases may support convenient access for health care professionals to search for and retrieve evidence-based medical information in clinical contexts [3,11,12]. Using evidence-based online medical databases to improve patient care is regarded as a legitimate clinical role of health care professionals [11]. Information literacy, that is, awareness and evaluation of the evidence sources, is also regarded as a necessary competency of health professionals in evidence-based practice [13].

### Information-Searching Behaviors

In spite of the help that web-based medical information provides in clinical decision making, its quality and credibility is an issue of concern, thereby suggesting the need to browse medical databases such as PubMed to cross-check web-based information and retrieve the best evidence [14]. Retrieving and applying information from evidence-based biomedical websites in clinical contexts is essential for health care professionals to improve patient care [2,15]. Most nurses and physicians (93%) who used evidence-based online medical databases believed that their use may help improve patient care [11]. Physicians with high usage of authoritative information sources, including scientific web-based databases such as PubMed and scientific digital journals, possess high fulfilment in meeting information needs and perceive themselves as having medical practice competency [7]. Clinicians’ use of evidence-based electronic knowledge resources has positive effects on their behaviors and on patients’ outcomes [16].

Although a large proportion of nurses use Google (80.2%), thereby making Google a regularly utilized electronic information resource, they seldom use web-based medical databases such as MEDLINE (19.8%) and Cochrane (1.1%) [9]. Moreover, there is a tendency for physicians to use nonauthoritative information sources such as Wikipedia, Facebook groups, and YouTube when retrieving web-based medical information [7]. Even though a majority of the health care professionals positively value the promotion of evidence-based medicine, they may not be aware of evidence-based medical information available online and may lack the ability to access validated evidence-based resources [3,17]. Further, medical students adopt less sophisticated information-searching strategies than their counterparts in general universities, thereby suggesting that additional training in searching for information on the internet is warranted [18].

Regarding the demographics of evidence-based online medical database users, the more senior the health care professionals are, the less likely they are to access evidence-based medical information on the internet [17]. Moreover, nurses require training in evidence-based practice, in particular, senior nurses with work experience of more than 5 years [19]. The age and educational level of physicians and nurses are also considered as important factors in predicting their usage of evidence-based online medical databases [11,20]. Regarding gender, male physicians are more likely than female physicians to employ web portals to search for medical information [20]. Thus, there are associations between the usage of internet-based medical resources and user characteristics such as gender, work experience, and academic degree [17,19,20].

### Epistemic Beliefs and Medical Information Searching on the Internet

Epistemic beliefs, a construct with multiple dimensions relating to the nature of knowledge and the way of knowing, have been defined as personal cognitions, including certainty of knowledge, simplicity of knowledge, source of knowledge, and justification for knowing [21]. Understanding how medical learners perceive and acquire medical knowledge (ie, their epistemic beliefs about medicine) has been considered an important issue in medical education [22,23]. Examining the relationship between epistemic beliefs and medical learning has potential for improving medical education [23]. Additionally, epistemic beliefs are regarded as an influential factor in searching on the internet [24,25]. While solving ill-structured problems through web-based information searching, the activation of epistemic beliefs may help learners who possess complex epistemic beliefs to use advanced search strategies, evaluate information quality, and search for alternative views [24]. Research on epistemic beliefs has focused on its role in processing diverse and conflicting information on the internet [26-28].

With respect to medical information searching, epistemic beliefs have also been defined as a determinant factor in dealing with controversial medical information and making health-related decisions [25,29-31]. Research on the navigation behaviors of adults without a university education showed that while searching for health-related issues on the internet, beliefs regarding justification for multiple resources and the reliability of web-based knowledge were positively related to the time spent on the objective webpages and recommended postsearch health decisions [31]. However, the lack of research on the influences of epistemic beliefs on health care professionals’ medical information–searching behaviors needs to be noted and explored.

Owing to the context-sensitive nature of personal epistemology, it has been suggested that beliefs about knowledge and knowing should be investigated in a specific context (eg, web-based searching) and measured with a context-specific instrument [27,28]. To evaluate epistemic beliefs in an internet-based environment, the construct and measurement of internet-specific epistemic beliefs have been developed to assess individual beliefs regarding internet-based knowledge and knowing [32]. In addition, the construct validity of internet-specific epistemic beliefs measurement has been rigorously examined and its relationships with web-based information search activities have been extensively explored [33-35]. Based on the four-dimension theory of personal epistemology developed by Hofer and Pintrich [21], the dimensions of the internet-specific epistemic beliefs questionnaire (ISEQ) were originally constructed by Bråten and his colleagues [32]. Additionally, the ISEQ was utilized to examine the roles of internet-specific beliefs in web-based health information searching behaviors and web search activities on a medical issue [31,35].

The Chinese version of the internet-specific epistemic beliefs questionnaire (C-ISEQ), adapted from ISEQ and translated into Chinese, was utilized to measure university students’ beliefs regarding internet-based knowledge and knowing in Taiwan [33]. The C-ISEQ has been rigorously modified and validated as having acceptable reliability and appropriate validity, denoting a four-dimension model with 12 items related to beliefs in certainty of internet-based knowledge, simplicity of internet-based knowledge, source of internet-based knowledge, and justification for internet-based knowing [33,34]. In addition, the C-ISEQ has been utilized to examine the relationships between internet-specific epistemic beliefs and web-based information-searching behaviors for course-related questions [33,36].

There are disciplinary differences in epistemic beliefs across domains [37,38]. For example, students of science and psychology have different views on certainty, source, justification, and truth of knowledge [37]. Furthermore, the domain specificity of epistemic beliefs has been referred to and explored, thereby showing the influential role of scientific epistemic beliefs in learning science [39,40]. With respect to medical education, medical students’ medicine-related epistemic beliefs may be related to their approach to learning medicine [22]. Presumably, when investigating web-based search behaviors in the context relating to a specific domain, there is a need to consider individuals’ epistemic beliefs in terms of their context specificity (eg, internet-specific) and their domain specificity (eg, medicine-related) simultaneously. Owing to a deficiency of epistemic beliefs measurement for both internet-based and medicine-related contexts, there is a need to develop an instrument to measure the internet-specific epistemic beliefs in medicine (ISEBM).

### Research Objectives

Clinicians’ lack of intention to access evidence-based medical information sources remains a challenge [13,17]. The behaviors of health care professionals in searching web-based evidenced resources and evaluating the reliability of the retrieved information have become an issue of concern and have been increasingly examined [3,12,13,41]. Based on the help of advanced epistemic beliefs in skilled search strategies and valid information evaluation [28,42], there may be a presumable relationship between health care professionals’ epistemic beliefs and their intention to search for evidence-based medical information in web-based biomedical databases. Considering the context, as well as the domain specificity of web-based medical information searching [32,38], the first purpose of this study was to modify and validate a measurement to assess internet-specific and medicine-related epistemic beliefs, which have not been previously researched. Previous studies targeted laypeople rather than health care professionals in exploring the relations between internet-specific epistemic beliefs and web-based medical information searching activities [31,35]. To the best of our knowledge, no research has focused on health care professionals’ epistemic beliefs in medicine and their relationship with evidence-based medicine. Therefore, the second purpose of this study was to explore the relationship between health care professionals’ ISEBM and their intention to use evidence-based online medical databases such as MEDLINE and Cochrane while retrieving medical information on the internet. In sum, the main research questions of this study are as follows:

1. Is the instrument utilized in this study valid and reliable for measuring health care professionals’ beliefs regarding internet-based medical knowledge and knowing?

2. What are the relationships between health care professionals’ internet-specific beliefs about medicine and their intention to utilize evidence-based online medical databases?

## Methods

### Recruitment

In 1 university-affiliated teaching hospital in Taiwan, this study purposefully recruited health care professionals with work experience of more than 6 months. All the participants in this study voluntarily responded to the survey. Before answering the questionnaire, they read the cover statement that stated the confidential nature of this survey and were informed that they were free to withdraw from the study. The return of the finished questionnaire was regarded as their consent to participate.

### Measures

#### ISEBM

Considering the same language and the cultural context, the C-ISEQ was adopted from the previous work of Chiu et al to develop the major measure of this study, namely, the ISEBM questionnaire [33]. In addition, the items of internet-specific epistemic beliefs developed in the work of Bråten et al were retrieved and included in the ISEBM questionnaire [32]. According to the suggested process, the questionnaire development was conducted in the following steps [43]. First, the construct of internet-specific beliefs was defined and discussed before the development of the questionnaire to ensure the content validity of the questionnaire. Then, the wording of each item of the ISEBM was carefully modified to assess individuals’ internet-specific epistemic beliefs about web-based medical knowledge and knowing. Next, the ISEBM was checked by experts to confirm the face validity. Finally, the statistical estimates of reliability and validity were calculated via the use of the structural equation modeling (SEM) technique to examine the validation of the ISEBM.

Originating from the theoretical constructs of C-ISEQ [33], the ISEBM questionnaire was constructed with 4 dimensions, namely ISEBM-CE (certainty), ISEBM-SP (simplicity), ISEBM-SO (source), and ISEBM-JU (justification). With regard to the domain-specific nature of epistemic beliefs, the specificity of domain knowledge should be considered while developing the measurement of epistemic beliefs in certain domains [38]. To take the specificity of medicine into consideration, a total of 18 items of internet-specific epistemic beliefs retrieved from prior studies [32,33] were revised with wordings to specify the internet-specific epistemic beliefs relating to web-based searching for medical information. As suggested by researchers, the face validity should be initially established when using borrowed measurements to develop a new instrument [43]. Prior to the analysis of construct validity through the statistical technique, 2 experts in medicine and information education evaluated the content and meaning of each item and asserted the face validity of ISEBM.

The four-dimension ISEBM consists of 18 items measured with a 5-point Likert scale ranging from 1 (strongly disagree) to 5 (strongly agree). The higher scores of certainty, simplicity, and source dimension represent the more naïve beliefs in the internet-based knowledge, that is, the respondents are more likely to believe that the knowledge retrieved from the internet is certain, simple, and accurate. On the contrary, higher scores on the justification dimension denote that the respondents possess the sophisticated belief that the internet-based knowledge claims should be carefully justified. The concepts of each dimension are illustrated in the following paragraph.

The certainty of internet-based knowledge in medicine measures beliefs that the medical knowledge found on the internet is certain and true. Respondents with high scores on certainty, indicating naïve beliefs, are less likely to doubt the certainty of medical knowledge found on the internet. Simplicity of internet-based knowledge in medicine assesses beliefs that the internet-based medical knowledge is simple and specific. A high score on simplicity implies the naïve view that the internet contains simple and detailed medical knowledge. Source of internet-based knowledge in medicine evaluates beliefs that the internet is a good source that offers correct and essential medical knowledge. Respondents with high scores, showing naïve views on source, are more likely to believe that the internet contains good and accurate medical knowledge. Justification for internet-based knowing in medicine examines the extent to which respondents believe that the medical claims on the internet should be evaluated and justified. Respondents with high scores on justification, demonstrating sophisticated epistemic views, believe that the internet-based medical claims should be critically evaluated against other sources.

#### Demographic Variables

In addition to the ISEBM questionnaire, the participants’ gender, years of working experience, and academic degree were included in the structural model of SEM and were regarded as independent variables. The female participants were treated as the reference group and coded as 0, while the male participants were coded as 1. Years of work experience was treated as a continuous variable, indicating the actual years of clinical work experience. Academic degree was coded as a 2-type categorical variable, including bachelor’s degree level (coded as 1) and master’s degree level (coded as 2). Regarding the dependent variable, intention to use evidence-based online medical databases was a single item measured with a 5-point Likert scale ranging from 1 (impossible) to 5 (extremely possible). It was retrieved and modified from prior studies, which investigated health care professionals’ willingness to perform web-based learning activities [44]. While answering this item, the participants indicated the extent to which they may employ the evidence-based online medical databases such as MEDLINE, Cochrane, and UpToDate when retrieving web-based medical information to answer medical problems in clinical contexts.

### Statistical Analysis

With respect to the participants’ demographics, descriptive analyses, cross-tabulation analysis, and one-sided *t* test analyses were conducted using the software SPSS version 22 (IBM Corp). Following the guidelines of the SEM analytic approach recommended by Hair et al [45], the measurement model of the ISEBM instrument and the structural model of the research hypotheses were examined via partial least squares-structural equation modeling (PLS-SEM) analysis. The PLS-SEM model was sequentially analyzed and interpreted in 2 stages. In the first stage, the factor loadings, composite reliability, average variance explained, and the Fornell-Lacker criterion [46] for the measurement model of the ISEBM instrument were evaluated to test the reliability and validity of the instrument. Following the two-step procedure recommendation, some measure items with inadequate estimates were deleted to reach an acceptable model fit [47]. In the second stage, the structural model (ie, path correlation analysis) was assessed to examine the relations among participants’ demographics (ie, work experience, gender and academic degree), ISEBM, and intention to use evidence-based online medical databases. The software SmartPLS3 was utilized to perform the PLS-SEM analyses. *P* values less than .05 were regarded as significant loadings and statistically significant relationships between variables.

## Results

### Participants

This study adopted the paper-and-pencil survey approach. The data were collected from March to June 2018. After deleting 4 cases with major missing values, the data from a sample of 273 health care professionals with clinical work experience of more than 6 months in one university-affiliated teaching hospital was employed in the following calculation. The group comprised 84 physicians, 45 nurses, 57 pharmacists, 63 therapists, 18 medical technologists, and 6 nutritionists. Among them, 172 (63.0%) were females and 101 (36.9%) were males. Their average age was 29.57 years (range 20-65 years) and their average work experience was 4.60 years (range 0.5-37 years). Regarding their academic degrees, 50 participants possessed a master’s degree, while 223 held a bachelor’s degree. All participants included in this study voluntarily responded to the survey. Moreover, informed consent was obtained from the participants. With respect to further analysis of the participant data, please refer to the statistical analyses reported in Multimedia Appendix 1.

### PLS-SEM Analysis of the Measurement Model

After deleting 6 items with loadings smaller than 0.5 and 1 item with a loading higher than 0.95, the 4-dimension model containing 11 items with significant loadings ranging from 0.72 to 0.92 was identified as a reasonable measurement model. The composite reliability values ranged from 0.89 to 0.94, indicating that the reliability was fairly good (larger than 0.7). Further, the average extracted values ranged from 0.70 to 0.84, suggesting acceptable convergent validity (larger than 0.5) [48]. Table 1 represents the details of the measurement items, factor loadings, reliability, and validity indices. In addition, as shown in Table 2, based on the Fornell-Lacker criterion, the square root of average variance explained for each factor was higher than the corresponding interfactor correlations ranging from 0.02 to 0.62, showing evidence of adequate discriminant validity [46,48].

**Table 1:** Results of loadings, reliability, and convergent validity analysis.

Items, subitems			Loading		Composite reliability		Average variance explained		Rho value		*α* value
**ISEBM-CE^a^**					0.89		0.81		0.77		.76
	ICE1. I could find accurate answers to medical problems on the internet.	0.88									
	ICE2. I am most confident that I have understood medical problems when I have used the internet as a source of medical information.	0.91									
**ISEBM-SP^b^**					0.94		0.83		0.90		.90
	ISP1. The internet provides abundant details about medical topics.	0.90									
	ISP2. The internet offers simple and specific knowledge regarding medical topics.	0.92									
	ISP3. The internet includes a lot of specific information related to medical issues.	0.91									
**ISEBM-SO^c^**					0.87		0.70		0.87		.82
	ISO1. Most medical information can be found on the internet.	0.71									
	ISO2. The internet involves various sources, which provide the correct answers to medical questions.	0.87									
	ISO3. The internet contains information sources offering most medical knowledge.	0.91									
**ISEBM-JU^d^**					0.94		0.84		0.93		.91
	IJU1. I would compare information from various sources to evaluate the trustworthiness of medical knowledge retrieved from the internet.	0.92									
	IJU2. I would judge the logicality of the medical knowledge that I find on the internet.	0.91									
	IJU3. For the same topic, I would check more sources to evaluate medical knowledge available on the internet.	0.92									

^a^ISEBM-CE: certainty of internet-specific epistemic beliefs in medicine.

^b^ISEBM-SP: simplicity of internet-specific epistemic beliefs in medicine

^c^ISEBM-SO: source of internet-specific epistemic beliefs in medicine.

^d^ISEBM-JU: justification of internet-specific epistemic beliefs in medicine.

**Table 2:** Results of the discriminant validity analysis.^a^

Factors	ISEBM-CE^b^	ISEBM-SP^c^	ISEBM-SO^d^	ISEBM-JU^e^
ISEBM-CE	*0.90*	—^f^	—	—
ISEBM-SP	0.41	*0.91*	—	—
ISEBM-SO	0.62	0.39	*0.84*	—
ISEBM-JU	0.02	0.49	0.18	*0.92*

^a^The correlations between factors are below the diagonal, while the square root values for average variance explained estimates (in italics) are presented on the diagonal.

^b^ISEBM-CE: certainty of internet-specific epistemic beliefs in medicine.

^c^ISEBM-SP: simplicity of internet-specific epistemic beliefs in medicine.

^d^ISEBM-SO: source of internet-specific epistemic beliefs in medicine.

^e^ISEBM-JU: justification of internet-specific epistemic beliefs in medicine.

^f^Not applicable.

### Path Correlations of the Structural Model

The structural model, combined with the measurement model of ISEBM, intention to use evidence-based medical databases, and demographic variables, including years of work experience, gender, and academic degree, was analyzed with PLS-SEM to evaluate the path coefficients between the variables. The values of variance inflation factor for independent variables ranged from 1.03 to 1.87, showing that there was no problem of collinearity [48]. In addition, the PLS-SEM results showed reasonable model fitness with root-mean-square error of approximation (RMSEA) of 0.071 [45].

**Figure 1:**
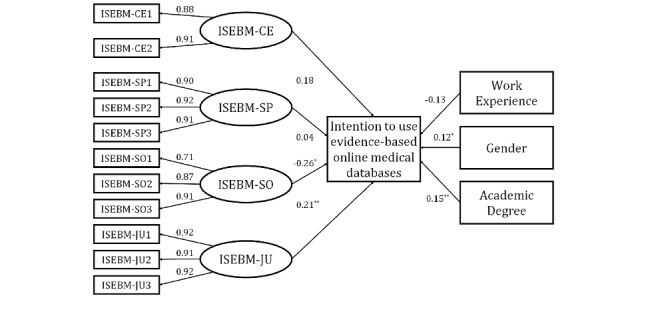
The partial least squares-structural equation modeling results for the measurement model and structural model. Intention indicates health care professionals’ intention to utilize the evidence-based online medical databases. With respect to gender, male is coded as 1 while female is coded as 0. Regarding academic degree, master’s degree is coded as 2 while bachelor’s degree is coded as 1. *P<.05, **P<.01. ISEBM-CE: certainty of internet-specific epistemic beliefs in medicine; ISEBM-SP: simplicity of internet-specific epistemic beliefs in medicine; ISEBM-SO: source of internet-specific epistemic beliefs in medicine; ISEBM-JU: justification of internet-specific epistemic beliefs in medicine.

The path coefficients are presented in Figure 1. The source of internet-based knowledge in medicine has a negative correlation (path coefficient –0.26, *P*=.01) between intention to use evidence-based online medical databases, while justification for internet-based knowing in medicine has a positive correlation (path coefficient 0.21, *P*=.001) with such intention. Regarding the demographics, gender (male) and academic degree (master’s degree) were positively correlated to such intention with coefficients of 0.12 (*P*=.04) and 0.15 (*P*=.004), respectively. In all, the R^2^ value for intention was 0.13, while the adjusted R^2^ value was 0.10.

## Discussion

### Principal Findings

This study advances the understanding of epistemic beliefs in web-based information-searching contexts and explored the role of internet-specific epistemic beliefs in utilizing evidence-based online medical databases. With respect to the medical issue in web-based searching contexts, there have been a small number of studies that have investigated the internet-specific beliefs of laypersons rather than those of medical professionals [31,33,49]. Due to the widespread utilization of the internet for searching for and locating medical knowledge in daily clinical practice [8,20], it is important to understand health care professionals’ internet-specific epistemic beliefs, which play an influential role in their web-based information-searching behavior [24,26]. Assisting them with effective usage of web-based resources is important to improve patient care [8].

### ISEBM and Intention to Use Evidence-Based Online Medical Databases

Individuals with sophisticated epistemic beliefs (rather than naïve beliefs) are more likely to use advanced information search strategies to evaluate the quality of web information by checking alternative sources [24]. In addition, beliefs regarding justification for multiple sources are positively related to deep web search activities [31]. As expected, the results of SEM with path analysis showed that naïve beliefs regarding the source of internet-based medical knowledge had a negative relationship with the intention to use evidence-based online medical databases, that is, those health care professionals who believed that the internet is a good source of accurate and essential medical information were less likely to search for evidences from evidence-based online medical databases. On the contrary, sophisticated beliefs regarding justification for internet-based knowing in medicine had a positive relationship with intention to use evidence-based online medical databases. In other words, health care professionals who held beliefs that there is a need to justify web-based medical information by checking alternative sources were more likely to employ evidence-based online medical databases.

Consistently, it was indicated that source and justification play an essential role in web-based information searching to explore an ill-structured task. Specifically, the credibility of an electronic source and the criteria for the justification of the knowledge were frequently referred to when participants epistemically reflected during their web-based searching [27]. In particular, the source of knowledge is the most epistemic reflection during the web-based information search for a controversial topic. Obviously, the arousal of individuals’ epistemic beliefs in source and justification is relatively important for improving their web-based searching behavior [24]. Accordingly, improving health care professionals’ internet-specific beliefs regarding credibility of source and rule of justification may inspire them to access evidence from web-based medical databases.

Previous studies have shown that promoting the searching skills for retrieving and evaluating web-based medical information is essential for clinical health care professionals [8,11,14,50]. Besides training in internet search skills, there is a crucial need to elevate health care professionals’ beliefs regarding internet-based knowledge and knowing in the medical domain. This study confirms the importance of improving epistemic beliefs to guide appropriate medical information behaviors [25,30]. Possessing advanced epistemic beliefs may stimulate information seekers to employ advanced strategies to evaluate the quality of web-based information [24]. Therefore, knowing health care professionals’ internet-specific beliefs can help medical educators improve their web-based medical information–seeking behaviors and assist them in evaluating and retrieving the best evidence for clinical decision making.

### Demographics and Intention to Use Evidence-Based Online Medical Databases

In addition to the influence of internet-specific epistemic beliefs on the intention to utilize the evidence-based medical databases, the demographics of health care professionals, including gender and academic degree, revealed a significantly influential role in the intention to search evidence-based online medical databases. Regarding the gender-related issue, gender differences in the use and perceptions of the internet remain a major concern when discussing web-based information-seeking behaviors [34,51]. Consistently, a gender difference was found in the intention to use web-based medical databases for evidence. Therefore, there is a need to investigate female health care professionals’ needs for web-based information and to help them locate appropriate information in relation to evidence-based medicine. Finally, in accordance with other research studies, the results of this study showed that academic degree was an influential factor in the intention to use evidence-based online medical databases, that is, participants with a master’s degree were more likely to search for evidence on medical databases. Perhaps their research training in their graduate courses gave them experience in searching web-based medical databases. Physicians’ additional research degrees and research practice activities are associated with their evidence-based medicine competency [5]. Therefore, improving the knowledge of published research evidence and increasing participation in research training may be of potential benefit to conducting web-based searching for evidence-based medicine [6,52].

### Limitations

This study has several major limitations that should be acknowledged. First of all, the generalizability of the study findings is limited. The participants surveyed were health care professionals in only 1 university-affiliated teaching hospital although it is a large-scale medical center. Second, the participants answered the paper-and-pencil questionnaire by themselves. Thus, self-reported bias may have occurred. Third, web-based medical databases are not the only reliable source of evidence-based medical information. Other alternative information sources of evidence-based medicine such as scientific, medical, health and nursing journals available in print cannot be underestimated and should be further studied. Fourth, the results of the structural model analysis showed a small R^2^ value of 0.13. In addition to the predictors included in the structural model shown in Figure 1, in future studies, there may be some other variables, which can be treated as predictors, moderators, as well as mediators in health care professionals’ intention to use evidence-based online medical databases. Finally, the RMSEA value of 0.071 revealed unsatisfactory model fit compared to the recommended threshold of 0.005 and below [53].

### Conclusions

Evidence-based medicine plays a determinant role in health care quality. The internet has been regarded as an evidence-based medical tool. Although the internet has become the most utilized resource for web-based medical information, health care professionals seldom access the validated evidence-based online medical databases. This study advances the knowledge on personal epistemic beliefs and their relationship with web-based information searching in clinical practice. Further, the results of this study provide suggestions for improving health care professionals’ intention to utilize the evidence-based online medical databases.

## Appendix

Multimedia Appendix 1 Statistical analyses of the participant data.

## Abbreviations

C-ISEQ: Chinese version of the internet-specific epistemic beliefs questionnaire
ISEBM: internet-specific epistemic beliefs in medicine
PLS-SEM: partial least squares-structural equation modeling
RMSEA: root-mean-square error of approximation
SEM: structural equation modeling

## References

[1] Sackett DL, Rosenberg WMC, Gray JAM, Haynes RB, Richardson WS (1996). Evidence based medicine: what it is and what it isn't. BMJ.

[2] Maggio LA, Kung JY (2014). How are medical students trained to locate biomedical information to practice evidence-based medicine? A review of the 2007-2012 literature. J Med Libr Assoc.

[3] Schuers M, Griffon N, Kerdelhue G, Foubert Q, Mercier A, Darmoni SJ (2016). Behavior and attitudes of residents and general practitioners in searching for health information: From intention to practice. Int J Med Inform.

[4] Al Omari Mousa, Khader Y, Jadallah K, Dauod AS, Al-Shdifat AAK (2009). Awareness, attitude and practice of evidence-based medicine among primary health care doctors in Jordan. J Eval Clin Pract.

[5] Buscaglia J, Nagula S, Yuan J, Bucobo JC, Kumar A, Forsmark CE, Draganov PV (2011). The practice of evidence-based medicine (EBM) in gastroenterology: discrepancies between EBM familiarity and EBM competency. Therap Adv Gastroenterol.

[6] Heighes Philippa T, Doig Gordon S (2014). Intensive care specialists' knowledge, attitudes, and professional use of published research evidence: a mail-out questionnaire survey of appropriate use of research evidence in clinical practice. J Crit Care.

[7] Mikalef P, Kourouthanassis PE, Pateli AG (2017). Online information search behaviour of physicians. Health Info Libr J.

[8] Clarke MA, Belden JL, Koopman RJ, Steege LM, Moore JL, Canfield SM, Kim MS (2013). Information needs and information-seeking behaviour analysis of primary care physicians and nurses: a literature review. Health Info Libr J.

[9] Sarbaz M, Kimiafar K, Sheikhtaheri A, Taherzadeh Z, Eslami S (2016). Nurses' Information Seeking Behavior for Clinical Practice: A Case Study in a Developing Country. Stud Health Technol Inform.

[10] Hornby K (2004). The internet as an evidence-based medicine tool. BCMedJ.

[11] Lialiou P, Mantas J (2016). Online Information Seeking Behaviour by Nurses and Physicians: A Cross-Sectional Study. Stud Health Technol Inform.

[12] Younger P (2010). Internet-based information-seeking behaviour amongst doctors and nurses: a short review of the literature. Health Info Libr J.

[13] Boruff JT, Harrison P (2018). Assessment of knowledge and skills in information literacy instruction for rehabilitation sciences students: a scoping review. J Med Libr Assoc.

[14] Hughes B, Joshi I, Lemonde H, Wareham J (2009). Junior physician's use of Web 2.0 for information seeking and medical education: a qualitative study. Int J Med Inform.

[15] Maggio LA, Tannery NH, Chen HC, Cate OT, O’Brien B (2013). Evidence-Based Medicine Training in Undergraduate Medical Education. Academic Medicine.

[16] Maggio LA, Aakre CA, Del Fiol G, Shellum J, Cook DA (2019). Impact of Clinicians' Use of Electronic Knowledge Resources on Clinical and Learning Outcomes: Systematic Review and Meta-Analysis. J Med Internet Res.

[17] Veness M, Rikard-Bell G, Ward J (2003). Views of Australian and New Zealand radiation oncologists and registrars about evidence-based medicine and their access to Internet based sources of evidence. Australas Radiol.

[18] Liang JC, Tsai CC (2009). The information commitments toward web information among medical students in Taiwan. Educational Technology & Society.

[19] Mokhtar IA, Majid S, Foo S, Zhang X, Theng Y, Chang Y, Luyt B (2012). Evidence-based practice and related information literacy skills of nurses in Singapore: an exploratory case study. Health Informatics J.

[20] Weng Y, Kuo KN, Yang C, Lo H, Shih Y, Chen C, Chiu Y (2013). Increasing utilization of Internet-based resources following efforts to promote evidence-based medicine: a national study in Taiwan. BMC Med Inform Decis Mak.

[21] Hofer BK, Pintrich PR (2016). The Development of Epistemological Theories: Beliefs About Knowledge and Knowing and Their Relation to Learning. Review of Educational Research.

[22] Chiu Y, Liang J, Hou C, Tsai C (2016). Exploring the relationships between epistemic beliefs about medicine and approaches to learning medicine: a structural equation modeling analysis. BMC Med Educ.

[23] Eastwood JL, Koppelman-White E, Mi M, Wasserman JA, Krug Iii Ernest F, Joyce B (2017). Epistemic cognition in medical education: a literature review. Int J Med Educ.

[24] Ulyshen TZ, Koehler MJ, Gao F (2015). Understanding the Connection Between Epistemic Beliefs and Internet Searching. Journal of Educational Computing Research.

[25] Knight S, Rienties B, Littleton K, Mitsui M, Tempelaar D, Shah C (2017). The relationship of (perceived) epistemic cognition to interaction with resources on the internet. Computers in Human Behavior.

[26] Mason L, Ariasi N, Boldrin A (2011). Epistemic beliefs in action: Spontaneous reflections about knowledge and knowing during online information searching and their influence on learning. Learning and Instruction.

[27] Mason L, Boldrin A, Ariasi N (2009). Searching the Web to learn about a controversial topic: are students epistemically active?. Instr Sci.

[28] Hofer BK (2004). Epistemological Understanding as a Metacognitive Process: Thinking Aloud During Online Searching. Educational Psychologist.

[29] Chua AY, Banerjee S (2017). To share or not to share: The role of epistemic belief in online health rumors. Int J Med Inform.

[30] Kienhues D, Stadtler M, Bromme R (2011). Dealing with conflicting or consistent medical information on the web: When expert information breeds laypersons' doubts about experts. Learning and Instruction.

[31] Kammerer Y, Amann DG, Gerjets P (2015). When adults without university education search the Internet for health information: The roles of Internet-specific epistemic beliefs and a source evaluation intervention. Computers in Human Behavior.

[32] Bråten I, Strømsø HI, Samuelstuen MS (2016). The Relationship between Internet-Specific Epistemological Beliefs and Learning within Internet Technologies. Journal of Educational Computing Research.

[33] Chiu Y, Liang J, Tsai C (2013). Internet-specific epistemic beliefs and self-regulated learning in online academic information searching. Metacognition Learning.

[34] Chiu YL, Tsai CC, Liang JC (2015). Testing measurement invariance and latent mean differences across gender groups in college students’ Internet-specific epistemic beliefs. AJET.

[35] Kammerer Y, Bråten I, Gerjets P, Strømsø Hi (2013). The role of Internet-specific epistemic beliefs in laypersons’ source evaluations and decisions during Web search on a medical issue. Computers in Human Behavior.

[36] Chiu Y, Liang J, Tsai C (2016). Exploring the roles of education and Internet search experience in students' Internet-specific epistemic beliefs. Computers in Human Behavior.

[37] Hofer BK (2000). Dimensionality and Disciplinary Differences in Personal Epistemology. Contemp Educ Psychol.

[38] Muis KR, Bendixen LD, Haerle FC (2006). Domain-Generality and Domain-Specificity in Personal Epistemology Research: Philosophical and Empirical Reflections in the Development of a Theoretical Framework. Educ Psychol Rev.

[39] Conley AM, Pintrich PR, Vekiri I, Harrison D (2004). Changes in epistemological beliefs in elementary science students. Contemporary Educational Psychology.

[40] Liang JC, Lee MH, Tsai CC (2010). The Relations Between Scientific Epistemological Beliefs and Approaches to Learning Science Among Science-Major Undergraduates in Taiwan. Asia-Pac.Educ.Res.

[41] Cullen RJ (2002). In search of evidence: family practitioners' use of the Internet for clinical information. J Med Libr Assoc.

[42] Bråten I, Brandmo C, Kammerer Y (2018). A Validation Study of the Internet-Specific Epistemic Justification Inventory With Norwegian Preservice Teachers. Journal of Educational Computing Research.

[43] Hair Jr JF, Black WC, Babin BJ, Anderson RE, Tatham RL (2006). Multivariate Data Analysis. 6th ed.

[44] Chiu Y, Tsai C (2014). The roles of social factor and internet self-efficacy in nurses' web-based continuing learning. Nurse Educ Today.

[45] Hair Jr. JF, Matthews LM, Matthews RL, Sarstedt M (2017). PLS-SEM or CB-SEM: updated guidelines on which method to use. IJMDA.

[46] Fornell C, Larcker DF (1981). Evaluating Structural Equation Models with Unobservable Variables and Measurement Error. Journal of Marketing Research.

[47] Anderson JC, Gerbing DW (1988). Structural equation modeling in practice: A review and recommended two-step approach. Psychological Bulletin.

[48] Hair Jr JF, Hult GTM, Ringle CM, Sarstedt M (2016). A Primer on Partial Least Squares Structural Equation Modeling (PLS-SEM).

[49] Kammerer Y, Gerjets P (2012). Effects of search interface and Internet-specific epistemic beliefs on source evaluations during Web search for medical information: an eye-tracking study. Behaviour & Information Technology.

[50] Scott SD, Gilmour J, Fielden J (2008). Nursing students and Internet health information. Nurse Educ Today.

[51] Bidmon S, Terlutter R (2015). Gender Differences in Searching for Health Information on the Internet and the Virtual Patient-Physician Relationship in Germany: Exploratory Results on How Men and Women Differ and Why. J Med Internet Res.

[52] Salbach N, Guilcher S, Jaglal S, Davis D (2009). Factors influencing information seeking by physical therapists providing stroke management. Phys Ther.

[53] Hu L, Bentler PM (1999). Cutoff criteria for fit indexes in covariance structure analysis: Conventional criteria versus new alternatives. Structural Equation Modeling: A Multidisciplinary Journal.

