# Supplementary material for: Internet-Specific Epistemic Beliefs in Medicine and Intention to Use Evidence-Based Online Medical Databases Among Health Care Professionals: Cross-sectional Survey
Source: J Med Internet Res. 2021 Mar 18;23(3):e20030. doi: 10.2196/20030 (PMC8074852; doi:10.2196/20030)
Supplement: Multimedia Appendix 1 [file jmir_v23i3e20030_app1.docx]

**Appendix:** Statistical analyses of the participant data

| **Cross tabulation analysis on gender and academic degree** | | | | |
| --- | --- | --- | --- | --- |
|  | | | Academic degree | |
| Gender, n (%) | | | Bachelor degree | Master degree |
|  | Female | 148(86) | | 24(14) |
|  | Male | 75(74.3) | | 26(25.7) |

*Note*: N = 273; Fisher’s exact test *X* ^2^ = 5.91, *P* = 0.023

| ***t*-test analysis on age** | | | | | |
| --- | --- | --- | --- | --- | --- |
| Academic degree | | N | Mean | SD | *t* value (*P* value) |
|  | Bachelor degree | 220 | 28.12 | 6.09 | -7.34 (*P* < .001) |
|  | Master degree | 48 | 36.21 | 9.91 |  |
| Gender | |  | | | |
|  | Female | 169 | 28.89 | 7.02 | -1.93 (*P* = .055) |
|  | Male | 99 | 30.73 | 8.35 |  |

*Note*: N = 268; SD = standard deviation

| ***t*-test analysis on work experience** | | | | | |
| --- | --- | --- | --- | --- | --- |
| Academic degree | | N | Mean | SD | *t* value (*P* value) |
|  | Bachelor degree | 223 | 3.89 | 5.06 | -3.09 (*P* = .003) |
|  | Master degree | 50 | 7.79 | 8.60 |  |
| Gender | |  | | | |
|  | Female | 172 | 4.55 | 5.76 | -0.18 (*P* = .856) |
|  | Male | 101 | 4.69 | 6.53 |  |

*Note:* N = 273; SD = standard deviation
